# Dataset and methodology on identification and correlation of secondary carbides with microstructure, wear mechanism, and tool performance for different CERMET grades during high-speed dry finish turning of AISI 304 stainless steel

**DOI:** 10.1016/j.dib.2020.105753

**Published:** 2020-05-21

**Authors:** Uttkarsh Patel, Sushant Rawal, A.F.M. Arif, Stephen Veldhuis

**Affiliations:** McMaster Manufacturing Research Institute (MMRI), Department of Mechanical Engineering, McMaster University, 1280 Main Street West, Hamilton, ON, L8S 4L7, Canada

**Keywords:** XRD, SEM, EDS, Phase identification, Tool Life, Wear mechanism, CERMET, Secondary Carbide

## Abstract

The aim of this research is to utilize reverse engineering approach for the identification of the elements and phases available in the commercial CERMET inserts with the help of characterization techniques such as Scanning Electron Microscope (SEM), Energy-dispersive X-ray spectroscopy (EDS), and X-Ray Deposition (XRD). Four commercial CERMET inserts were investigated in this research work, and the effect of the composition and phases are related to its tool wear mechanism and performance. Each CERMET insert is used to perform a turning process on a CNC lathe for machining stainless steel (SS) under the dry condition at a fixed cutting length interval. Once it completes machining for a fixed cutting length, the CERMET insert is taken out to investigate its wear mechanism with the help of SEM, EDS, XRD and using a focus-variation microscope (Alicona). A correlation analysis is performed to relate progressive tool wear mechanisms with elements and their relevant phases of various carbides. The approach of correlating wear property with the phase content will contribute to the understanding of the wear mechanism under such extreme machining conditions. It will serve as a reference for the improvement of the performance of these CERMET inserts for such harsh machining conditions by the development of protective coatings for these CERMET inserts based on the identification of the composition and phases that improves tool life and reduces wear. The data related research work can be found at “https://doi.org/10.1016/j.wear.2020.203285” [1].

**Specifications Table**

**Table:** 

**Subject**	Ceramics and Composites
**Specific subject area**	Influence of the composition and phases available in commercial CERMET inserts on its wear mechanism and tool life for machining stainless steel
**Type of data**	Table (Procedure steps for the polishing). Images (SEM images, and microscopic images). Graphs (EDS point spectra)
**How data were acquired**	The following experimental techniques were used to acquired data: Scanning Electron Microscope (SEM): - Using “JEOL 6610LV” for SEM images and Elemental identification (EDS). (ref. Fig. 1, Fig. 2, and Fig. 3) X-Ray Diffraction (XRD): - Bruker D8 discover with cobalt source. CNC turning lathe: - OKUMA crown L1060 for tool life test and tool wear test Digital Images: - KEYENCE-VHX 5000 digital microscope to capture tool wear from various orientation (ref. Fig. 4) 3D Scan Microscope: - Alicona Infinite Focus G5 microscope to capture 3D wear volume (ref. Fig. 5)
**Data format**	Raw data for microstructure SEM images, XRD, and EDS. Tool life with flank wear and notch wear are collected and prepared as graphs. Volume wear data for the CERMET inserts was measured by the software of Alicona Infinite Focus G5 microscope, analyzed and prepared as graphs.
**Parameters for data collection**	SEM: Electron beam: 20-25KeV Spot size: 55 WD: 10-12mm XRD: Cobalt sealed tube source (λ_avg_ = 1.79026Å) Power settings: 35kV, 45mA Scan at six steps in 22-112˚ range, Exposure time: 480s/step. CNC turning lathe: Cutting speed: 240m/min Depth of cut: 0.4mm Feed:0.15 mm/rev
**Description of data collection**	Four commercial CERMET inserts were investigated in this research work. Each new CERMET insert was scanned before start of the experiment to compare it later with its machined tool surface. The machining test was performed at a fixed cutting interval. The SEM images were captured to keep track of tool wear and to understand the tool microstructure, and EDS was performed at the fixed cutting interval to identify its composition. XRD was done to determine the relevant phases present, and a 3D scan was taken using a focus variation microscope (Alicona) to measure the wear volume after each cutting interval. A digital microscope was also utilized to measure flank wear and notch wear.
**Data source location**	SEM: (Fig. 1, Fig. 2, and Fig. 3) Institution: Canadian Centre for Electron Microscopy (CCEM) Facility, McMaster University City/Town/Region: A.N. Bourns Science Building, 1280 Main St W, Hamilton, ON, L8S 4M1 Country: Canada XRD: Institution: McMaster Analytical X-Ray Diffraction (MAX) Facility, McMaster University City/Town/Region: A.N. Bourns Science Building, 1280 Main St W, Hamilton, ON, L8S 4M1 Country: Canada Tool life: Institution: McMaster Manufacturing Research Institute (MMRI), McMaster University City/Town/Region: John Hodgins Engineering Building, 1280 Main St W, Hamilton, ON L8S 4L7 Country: Canada Digital microscope image & 3D scan images (Fig. 4 and Fig. 5) Institution: McMaster Manufacturing Research Institute (MMRI), McMaster University City/Town/Region: John Hodgins Engineering Building, 1280 Main St W, Hamilton, ON L8S 4L7 Country: Canada
**Data accessibility**	In a public repository: Repository name: [Mendeley Data] Data identification number: [10.17632/xg2rngb8xn.1] Direct URL to data: [10.17632/xg2rngb8xn.1]
**Related research article**	Author's name: Uttkarsh S. Patel, Sushant K. Rawal, A.F.M. Arif, Stephen C. Veldhuis Title: Influence of secondary carbides on microstructure, wear mechanism, and tool performance for different CERMET grades during high-speed dry finish turning of AISI 304 stainless steel [1]. Journal: Wear Volume: 452–453 Year: 2020 10.1016/j.wear.2020.203285

**Value of the Data**

• These data provides a comprehensive comparison and explanation on the effect of various tool compositions and phases on the wear mechanism and tool life for dry machining of stainless steel by four different commercial CERMET inserts.
• These data will provide a reference for the manufacturing sector on the identification and selection of appropriate CERMET insert based on tool life results for dry machining of stainless steel.
• These data will be used for the development of protective coatings for these CERMET inserts based on the identification of the composition and phases that improve tool life and reduces wear.
• These data can serve as a benchmark for CERMET insert to be explored for additional machining applications for different materials in similar dry machining conditions.

## 1 Data Description

**Fig. 1:**
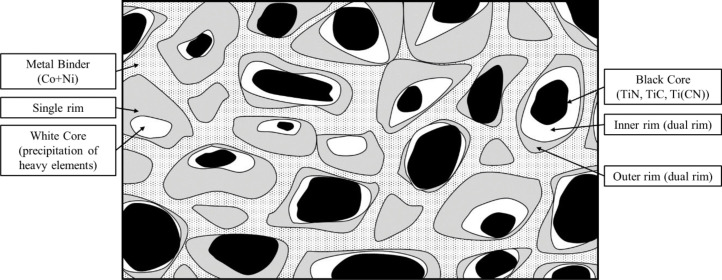
Backscattered electron (BSE) SEM images of all CERMET tools (a) Tool A, (b) Tool B, (c) Tool C, and (d) Tool D.

**Fig. 2:**
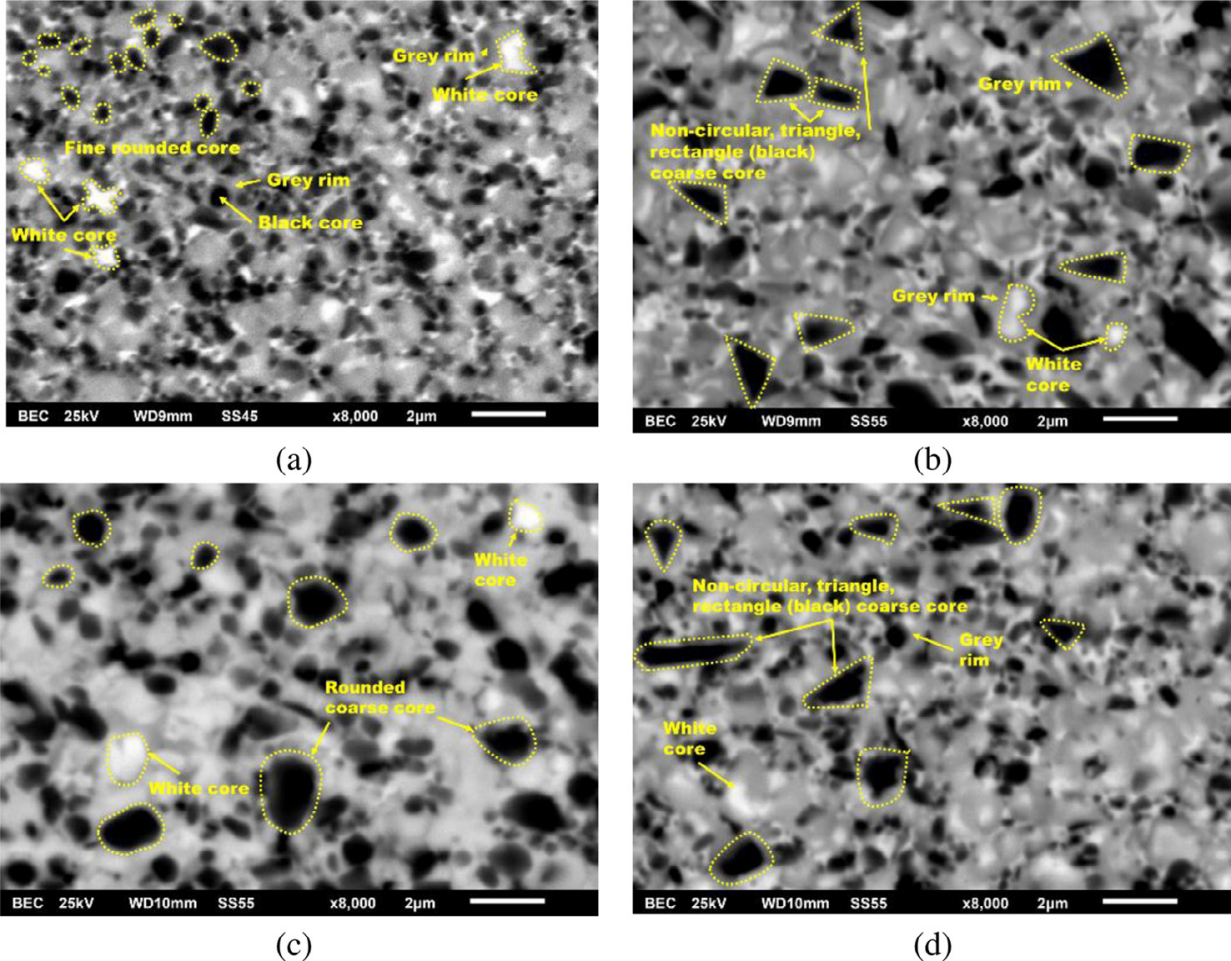
Secondary electron SEM images of all CERMET tools (a) Tool A, (b) Tool B, (c) Tool C, and (d) Tool D.

**Fig. 3:**
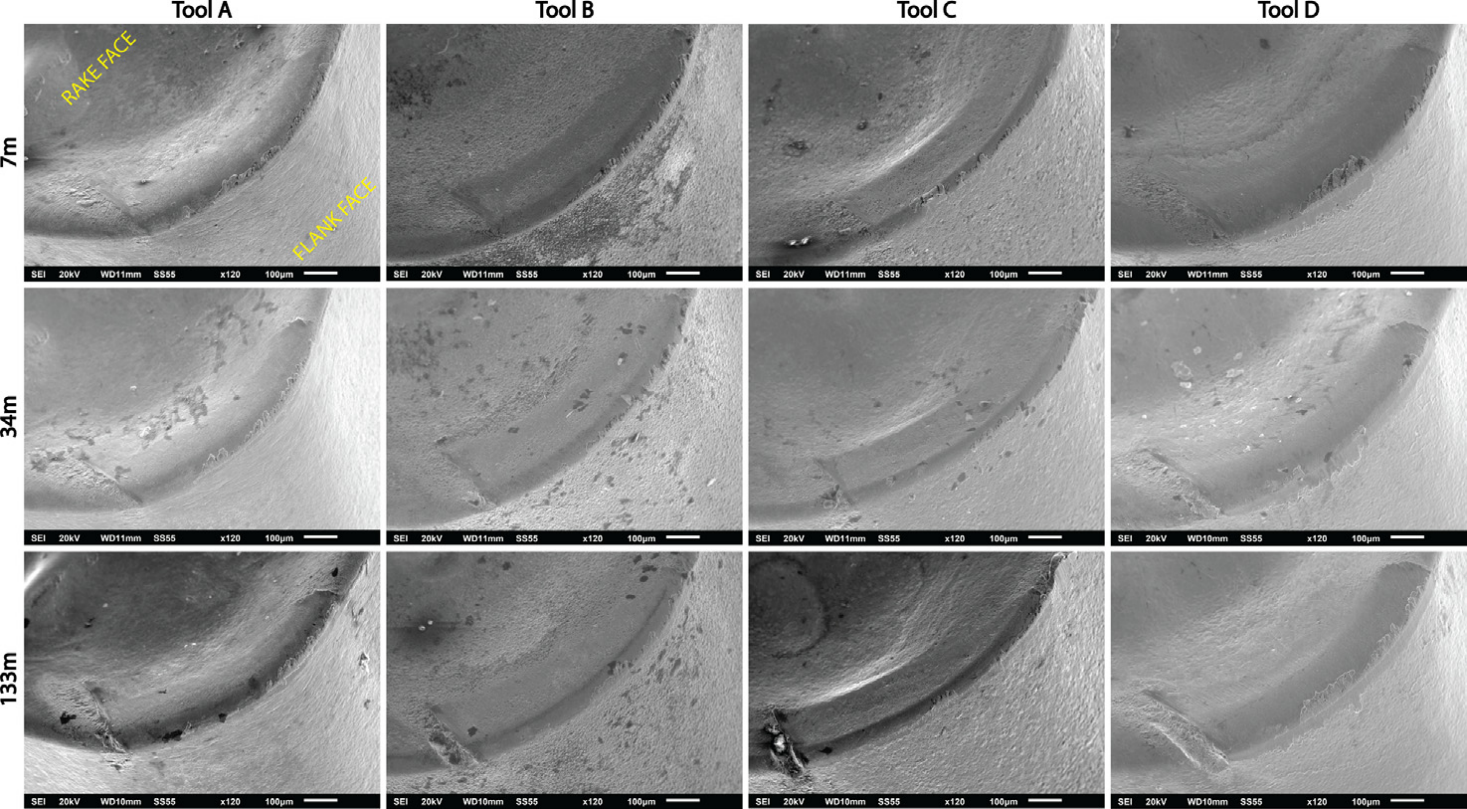
Secondary electron SEM images of all CERMET tools after machining lengths of 7m, 34m, and 133m showing microchipping and notch generation.

**Fig. 5:**
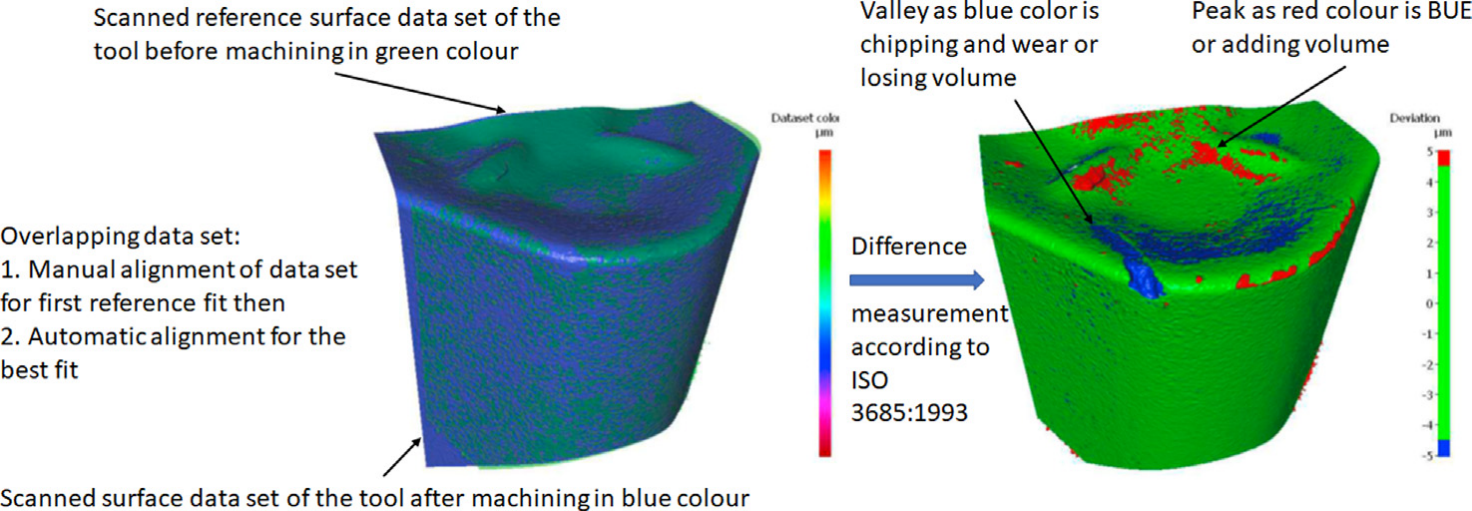
Illustration of the methodology used to identify three-dimensional wear volume.

Data presented in the article is on the comparison of wear mechanism and tool life for dry machining of stainless steel by four different commercial CERMET inserts. The experiments were performed to identify the various elements present in CERMET inserts, evaluate their effect on microstructure, and relate it to its wear mechanism and tool performance. In the first step, new CERMET inserts were used to capture microstructure (Figs. 1 and 2) and EDS results (Refer figure 5 of the article [1]). In the second step, an XRD test was performed to identify the phases related to these elements. The wt.% of major phases were calculated using the Topas software. A machining test was performed at a fixed interval to measure and quantify the tool wear under an optical microscope and a focus variation microscope (Alicona). The collected data was used to prepare a tool life graph. SEM images were also taken to investigate the progressive wear mechanism, as shown in Fig. 3. The 3D wear volume images were taken by scanning the CERMET insert with Alicona (Fig. 5) and presented as linear regression to compare the tool wear (Refer figure 10 of the article [1]).

## 2 Experimental Design, Materials, and Methods

### 2.1 Sample preparation for SEM

The samples were mounted in bakelite (hot mount) and polished with the help of auto polisher to make it flat and smooth mirror finished using the following steps as given in Table 1

**Table 1:** Steps to polish the CERMET inserts/coupons.

Step No.	Surface	Suspension	Lubrication	Time (min)
1.	Piano 120		Water	2:30
2.	Piano 600		Water	2:30
3.	Plan	DP-A 15μm	Blue	2:30
4.	Allegro	DP-A 9μm	Blue	4:00
5.	Dur	DP-A 3μm	Blue	4:00
6.	Dur	DP-A 1μm	Blue	6:00
7.	Chem	OP-S		2:00

Once samples were mirror polished, they were unmounted and placed on an SEM fixture for the SEM characterization.

### 2.2 Scanning Electron Microscopy (SEM) images

JEOL 6610LV SEM instrument was used to obtain the microstructure images of the CERMET inserts. The polished CERMET inserts were coated with the carbon/gold, and nickel paste was applied to create a path for the electron to pass through it so charging effect can be minimized and more clear and sharper image can be obtained. A backscattered electron (BSE) mode was used to capture chemical (atomic) contrast, which can help to identify core, rim and binder compounds in the CERMET inserts, as shown in Fig. 1.

To capture the surface topography, BSE mode was changed to secondary electron mode, and the same procedure as above was followed, and the images, as shown in Fig. 2, were obtained.

### 2.3 Energy-dispersive X-ray spectroscopy (EDS)

Polished CERMET inserts were loaded into the chamber and brought near to the detector until the working distance was 10-12mm. The EDS detector and software available to operate the equipment were used to collect X-rays at a minimum of 20KeV from the sample surface to excite X-rays from all the elements. The software was used to keep the dead time around 30%. The data was acquired and processed by the software to get the EDS spectrum (Refer figure 5 of the article [1]).

The investigation of elemental concentration (wt.%) of all CERMET tools by using JEOL 6610LV SEM device with the help of EDS which allows the user to identify the various elements and their concentration are summarized in Table 3 of paper [1].

### 2.4 X-Ray Diffraction (XRD)

Once the elements are confirmed, the same samples were used to perform the XRD for phase identification. The samples were mounted on the Bruker D8 discover with cobalt source having a wavelength of λ_avg_= 1.79026Å, and power settings: 35kV, 45mA. The data was collected through the scanning performed at 2θ values from 22° to 112° and at an increment of 18.

Software “DIFFRAC.EVA V4.2.1” was used to merge all frame and mask it to remove noise. A 1-dimension XRD spectra were created by using the tool “integrate cursor” and intensity v/s 2θ plot was generated. The “export partial scan” tool was used to flatten and export intensity plot by removing background noise.

The exported plot was saved as an XY file, and the data was used in the origin software to create a plot graph and stack multiple plots into a single graph for comparison (Refer figure 3 of the article [1]).

The same “DIFFRAC.EVA” software was used to search and identify phases from the database and match them with the XRD intensity plot data for all CERMET tools. In this case, ICDD PDF-4+ 2019 database was matched with CERMET tools, and peaks, along with its orientations for the identification of the respective phases (Refer figure 3 of the article [1]).

TOPAS software was used to measure the wt.% of phases identified by XRD. A CIFs file related to the phase for which we want to measure the wt.% is required and can be downloaded from the “ICSD Web-Inorganic Crystal Structure Database.” The CIFs files for the respective phases were downloaded and matched with the XRD raw data files using TOPAS, and the wt.% of phases results were summarized in Table 4 of the article [1].

### 2.5 Tool life and wear test

**Fig. 6:**
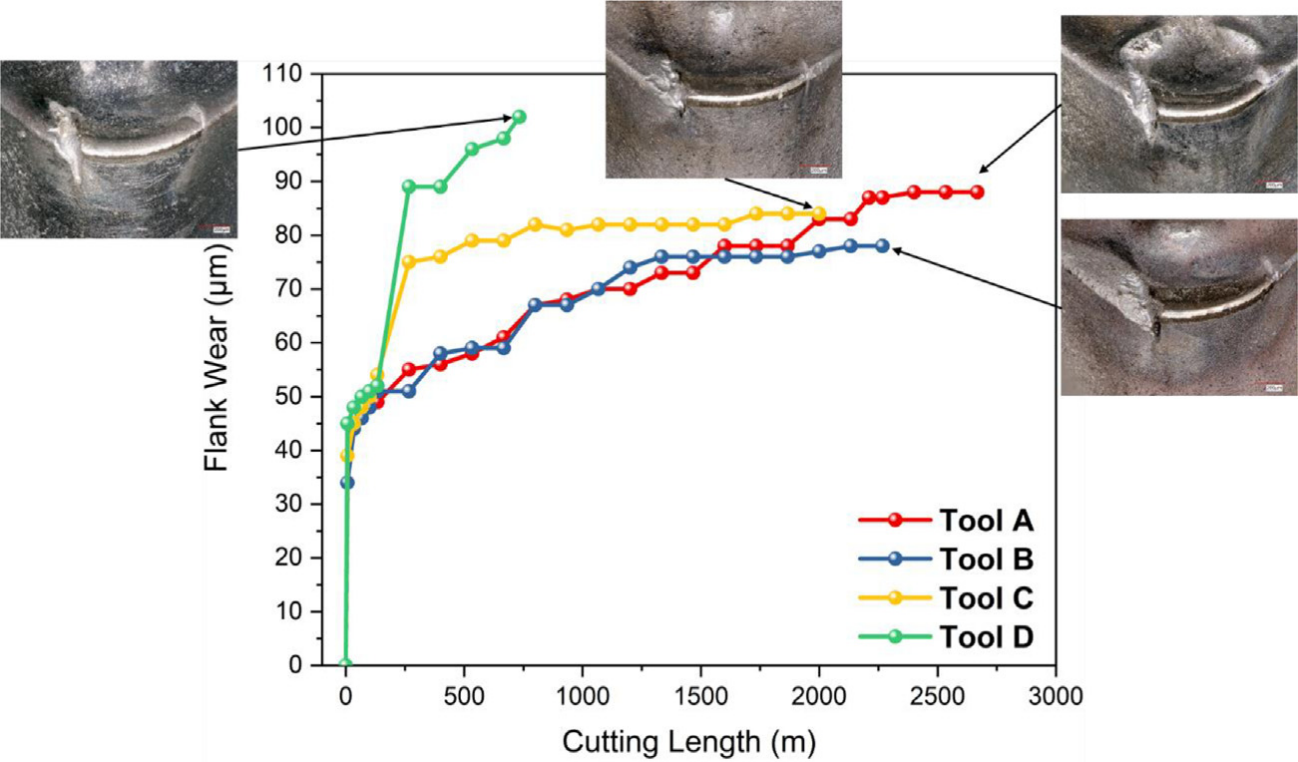
Tool flank wear progression with cutting length

**Fig. 7:**
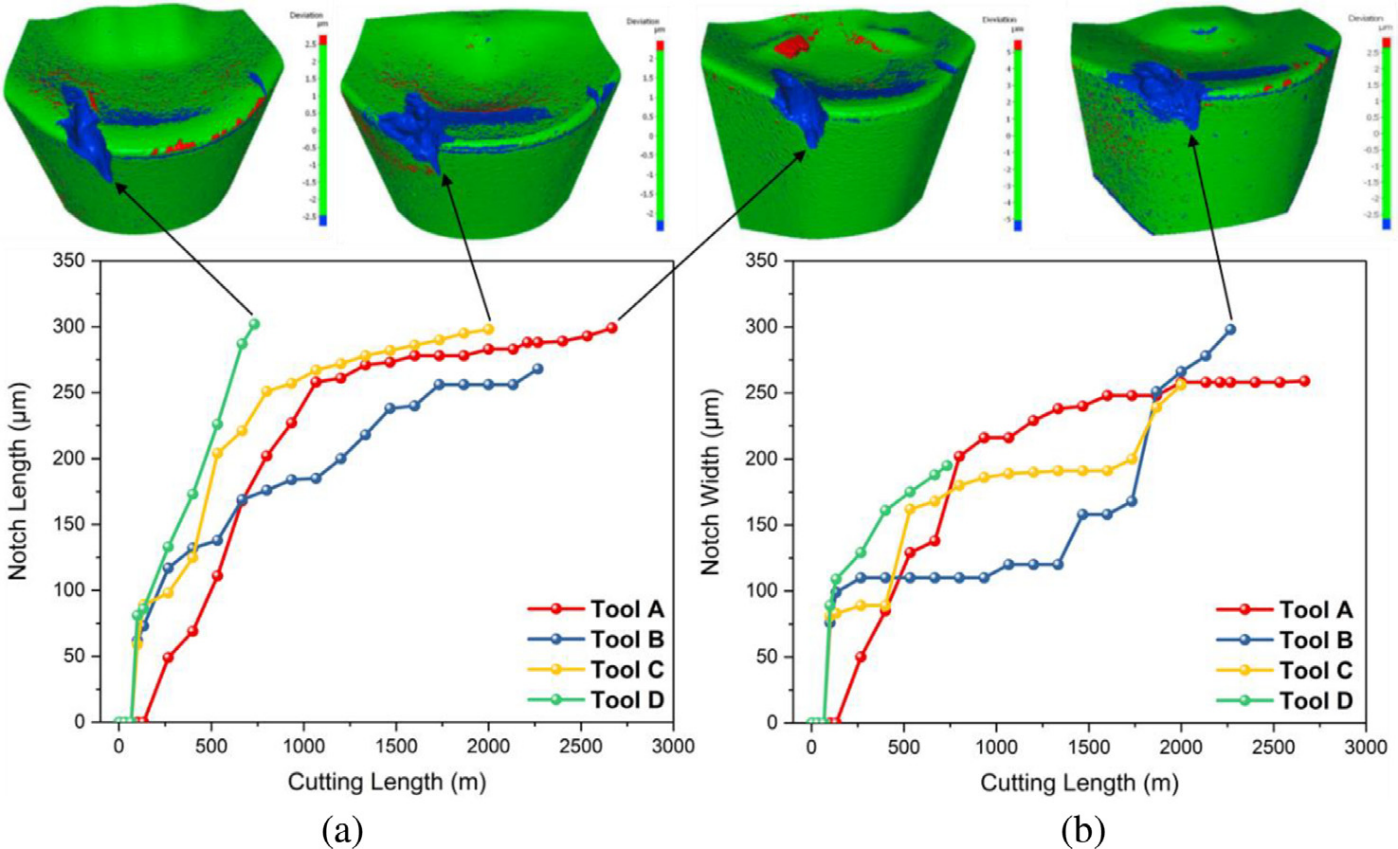
Tool notch wear progression with machining length: (a) notch length and (b) notch width

The tool life test for four different commercial uncoated inserts (CNMG 12408/CNMG 432) was performed using CNC OKUMA crown L1060 turning the machine on AISI 304 stainless steel workpiece. The feed rate of 0.15 mm/rev, depth of cut of 0.4mm and cutting speed of 240 m/min was used for the test. The machining was done for a fixed interval, and the inserts were examined for flank wear measurement along with notch length and width measurement. The data were measured and recorded in an MS Excel sheet, and the test was run until the tool reached the failure criteria of 300μm for either notch or flank wear. The collected data were processed in the origin software to prepare the tool life curves. The images collected at several intervals with the digital microscope (KEYENCE-VHX 5000 digital microscope) and focus variation (3D scan with Alicona Infinite Focus G5) were plotted on the same graph to get an idea about tool wear condition at a particular stage. The tool flank wear progression (Refer figure 6 of the article [1]) and the notch wear progression (Refer figure 7 of the article [1]) are shown with respect to the cutting length and discussed in article [1].

### 2.6 Progressive wear assessment

SEM examination was done at several intervals (7m, 34m, 67m, 100m, 133m, and after every additional 133m until failure of the tool) on all the four CERMET tools during the tool life test. The samples were mounted on the tool holder with 45˚degree inclined studs to track the wear progression, as shown in Fig. 3, as examined by SEM. The images were taken at 20KeV, at a spot size of 55 and a working distance of 11mm at a magnification of 120X.

**Fig. 4:**
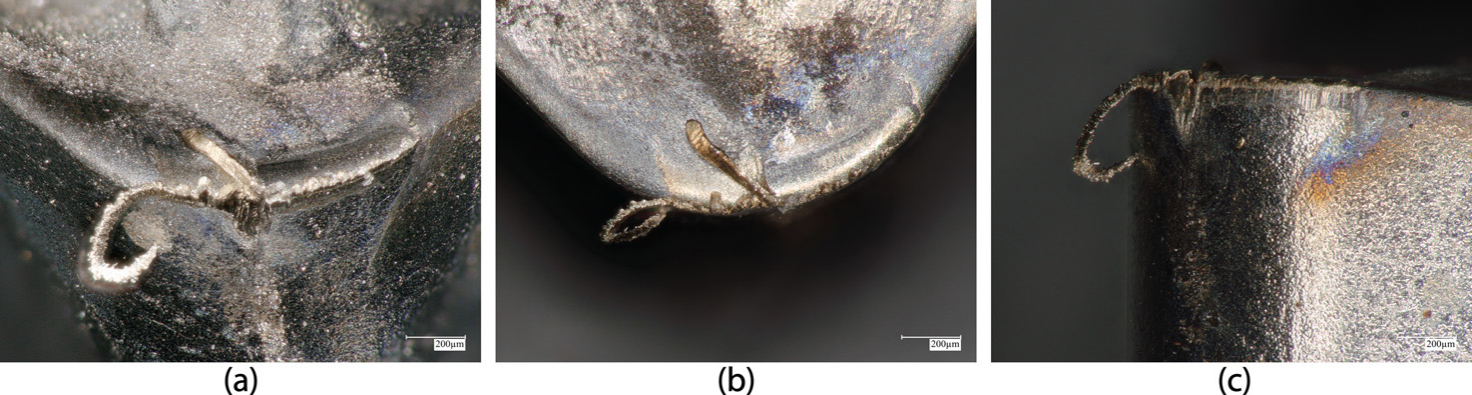
Optical microscope image of sticking and filling mechanism of workpiece material in the DOC notch groove formed on the CERMET tool by chipping (a) inclined view of notch groove where the workpiece material stick and form chip, (b) Rake view (c) side view or flank face view.

The CERMET tools were taken out of the CNC turning lathe and examined under the microscope without any cleaning or allowing the external interface to see the sticking material on the tools. The CERMET tools were examined under the digital microscope (KEYENCE-VHX 5000 digital microscope), and images were taken at a different angle to examine the tool and wear mechanism. Fig. 4 shows the sticking and notch filling mechanism with the workpiece material on the tools, as observed under an optical microscope.

### 2.7 3D wear volume measurement and comparison

**Fig. 8:**
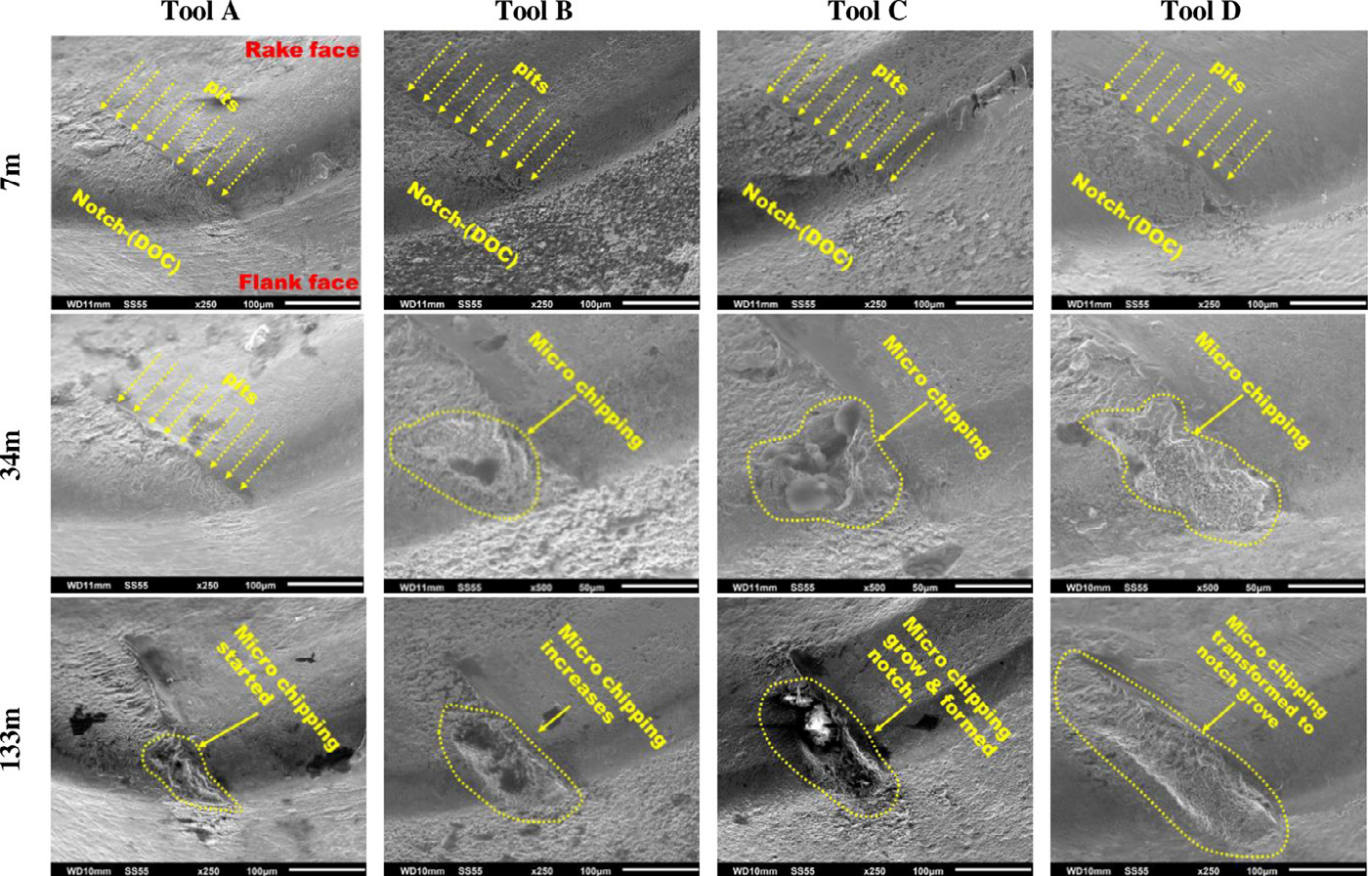
Secondary electron SEM images of all Ti(C,N)-based cermet tool after machining lengths of 7m, 34m and 133m showing microchipping and notch generation

**Fig. 9:**
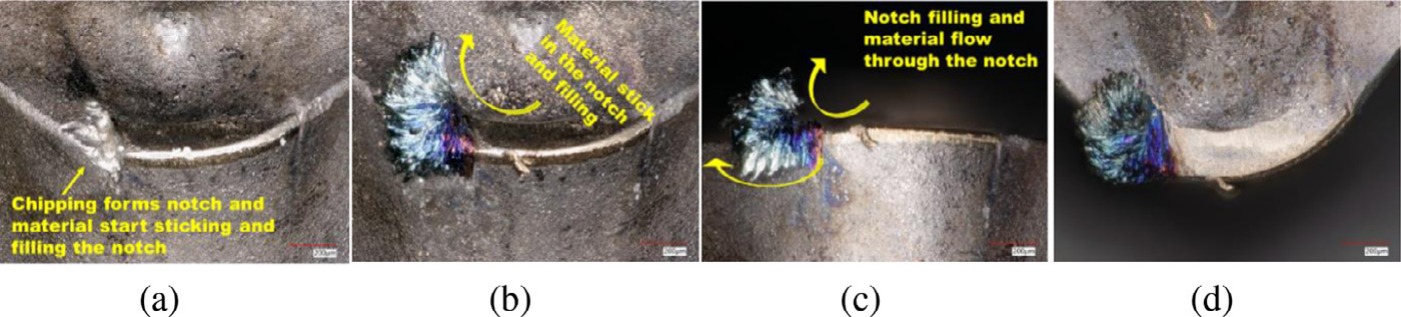
Sticking and filling mechanism of workpiece material in the DOC notch groove formed by chipping (a) notch grove where the workpiece material sticking, (b) sticking, filling, and sliding of workpiece material in the notch, (c) side view of the notch filling and sliding mechanism, (d) top view of the notch and insert

**Fig. 10:**
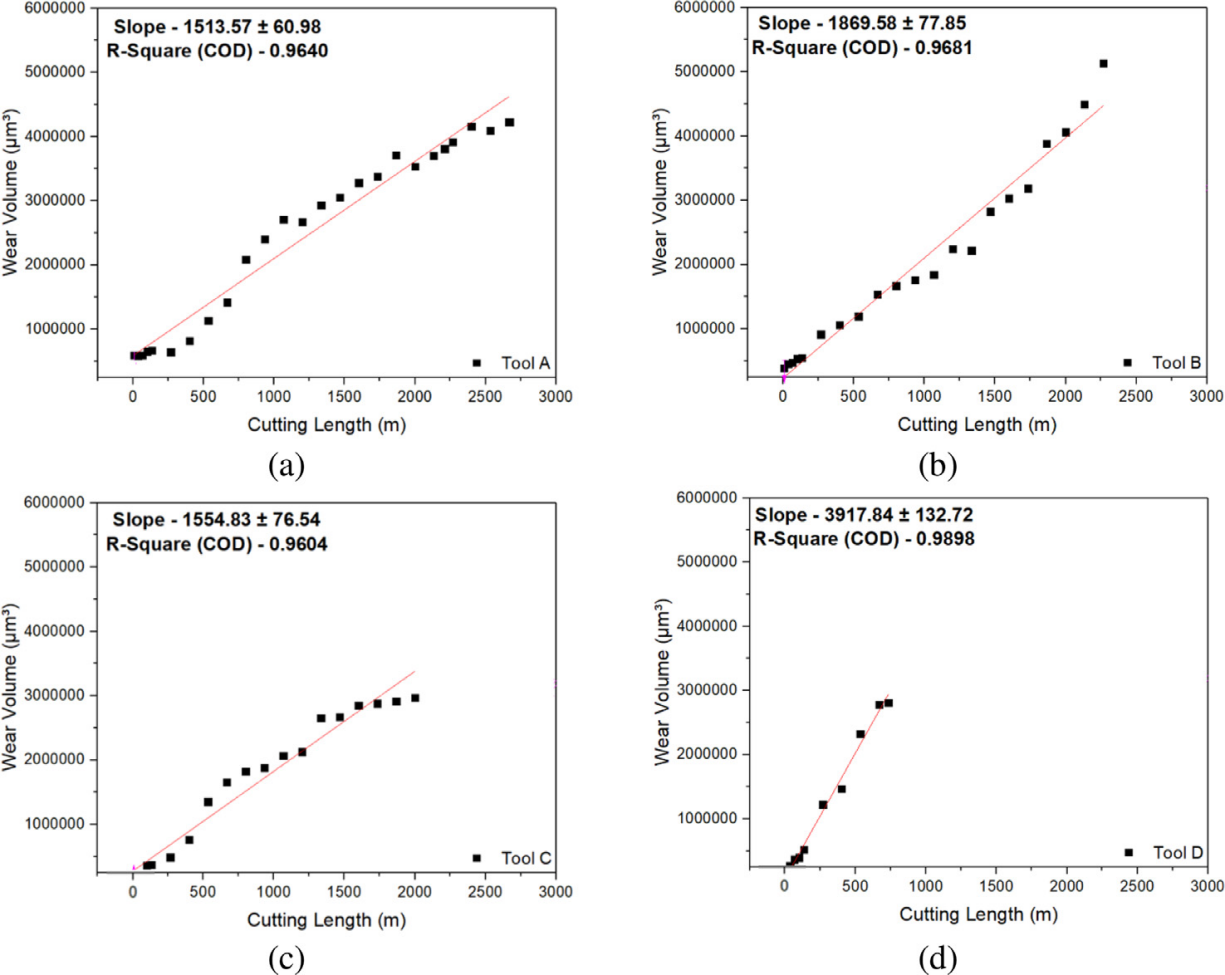
Linear regression of wear volume showing wear volume rate with machining length for (a) Tool A, (b) Tool B, (c) Tool C, and (d) Tool D.

The wear volume was measured using the Alicona Infinite Focus G5 microscope. First, the new CERMET tools before machining were scanned and examined with Alicona. The CERMET tools after every fixed interval during the machining test were scanned under Alicona again. The scanned CERMET tools were used to compare and calculate the wear volume with respect to new CERMET tools. By applying the proper alignment of two data sets, the software in Alicona shows the peaks and valleys with respect to the reference scan (new/fresh tool). The software then calculates the volume of the valley (wear, chipping) and peak (sticking of material, BUE), an example is shown in Fig. 5. The collected wear volume data was plotted with respect to cutting length, and linear regression analysis was performed with the help of origin software (Refer figure 10 of the article [1]).

## Declaration of Competing Interest

The authors declare that they have no known competing financial interests or personal relationships that could have appeared to influence the work reported in this paper.

## Appendix Supplementary materials

Image, application 1
